# Genetic Variation Disrupts RNA Structural Repression of Cryptic Splice Sites

**DOI:** 10.21203/rs.3.rs-11246309/v1

**Published:** 2026-10-07

**Authors:** Samuel Ogunsola, Jian Yang, Roby Joehanes, Daniel Levy, Chunyu Liu, Jiuyong Xie

**Affiliations:** 1 Department of Physiology & Pathophysiology, Max Rady College of Medicine, Rady Faculty of Health Sciences, University of Manitoba, Winnipeg, MB, R3E 0J9, Canada; 2 Department of Biostatistics, Boston University School of Public Health, Boston, MA, 02118, USA; 3 Framingham Heart Study, Framingham, MA 01702, USA; 4 Population Sciences Branch, Division of Intramural Research, National Heart, Lung, and Blood Institute, National Institutes of Health, Bethesda, MD 20892, USA

**Keywords:** G-tracts, RNA secondary structure, cryptic splice sites, single nucleotide variants

## Abstract

Interpreting how genetic variants alter RNA processing remains a major challenge in human genetics. Using Framingham Heart Study data and genome-wide and mutational analyses, we show that G-tract-disrupting variants activate cryptic 3′ splice sites in association with predicted local RNA structural destabilization, while compensatory mutations restoring structural stability re-establish splicing repression. Approximately 1.6 million such sites occur genome-wide, revealing RNA structural disruption as a candidate mechanism of variant-induced cryptic splicing.

Understanding the functional consequences of genetic variation, particularly in non-coding regions (NCRs), remains a major challenge in human genetics. Aberrant and cryptic splicing provides one route through which variants can alter gene function (1–5), yet many variant-associated cryptic splice sites are poorly explained by conventional splice-site sequence strength. We recently identified G-tracts immediately upstream of AG dinucleotides in human genes, forming G-tract-AG 3′ splice-site-like (3′SSL) signatures that differ from the pyrimidine-rich organization of canonical 3′ splice sites (3′SSs) (6–9) (Fig. 1A). These signatures are widespread in NCRs and can repress cryptic splicing (6). Their repressive activity depends on G-tract length and G-allele dosage and acts predominantly at the second transesterification step (6). Here, we ask whether genetic variants activate this class of cryptic splice sites by disrupting a local RNA structural state that is not captured by conventional splice-site scoring.

In genotype- and RNA-seq-matched Framingham Heart Study (FHS) data (6,10), G-tract-disrupting non-G alleles (A, C, or T; H-variants) were associated with significantly increased 3′AG usage (6), providing a population-based genetic effect on cryptic splicing. However, usage of these G-tract-AG sites did not correlate with their predominantly negative MaxEnt scores, a measure of primary splice-site sequence strength (11) (S_Fig. 1A). By contrast, usage of annotated 3′SSs in an independent dataset correlated strongly with their predominantly positive MaxEnt scores (S_Fig. 1B). The H-variant-associated increase in 3′AG usage likewise remained uncorrelated with MaxEnt scores (S_Fig. 1A), indicating that conventional splice-site sequence strength alone does not adequately explain these genetic effects.

We therefore examined predicted local RNA structural stability. 3′AG usage showed a stronger relationship with minimum free energy (ΔG) predicted by RNAfold (S_Fig. 1C). Analysis of 3,424 FHS participants further quantified the additive effect of H versus G alleles on 3′AG usage (Fig. 1B). Across the full range of ΔG values examined, H-variant effect size showed a significant correlation with ΔG in the more structurally stable range below approximately −4.0 kcal/mol (left panel) but remained uncorrelated with MaxEnt scores (right panel). This empirically observed transition was subsequently used as a threshold for genome-wide comparisons. Thus, predicted local structural stability is particularly informative for a subset of variant-induced cryptic splicing events that are not well explained by primary splice-site sequence strength.

Genome-wide analyses supported this distinction between primary splice-site sequence strength and predicted local RNA structural stability. Compared with annotated human 3′SSs, G-tract-AG 3′SSLs identified in human genes were enriched for negative MaxEnt scores and for sequences predicted to form stable local structures (Fig. 1C, MaxEnt < 0: 84% versus 4%; ΔG < −4 kcal/mol: 27% versus 12%; *p* < 1 × 10^−300^ for each comparison). Moreover, G-tract-disrupting sQTL variants also preferentially increased ΔG (Fig. 1D). In silico replacement of G-tracts with A-, C-, or U-tracts increased ΔG at 82%, 69%, and 77% of these sQTL sites, respectively. These observations indicate that G residues frequently contribute to predicted local structural stability at G-tract-AG 3′SSLs, including those associated with sQTLs. However, since both MaxEnt and ΔG are sequence-derived measures, sequence and structural contributions cannot be fully separated computationally.

To test whether structural destabilization can mediate a genetic effect on splicing, we performed mutagenesis and compensatory-mutation analyses. In a reporter for rs762810-dependent *MAX* exon 4 inclusion (6), the wild-type (G)_4_ sequence is predicted to form a stem-loop between the branchpoint region and 3′AG and is associated with nearly complete exon skipping (Fig. 2A–C, R1). The rs762810-T variant or replacement of the G-tract with an A-tract destabilized the predicted structure and increased exon inclusion (R2-R3). Critically, compensatory mutations that restored predicted structural stability without restoring the original G-tract sequence reestablished repression (R4-R5). Similar structure-associated changes occurred in additional reporter contexts (S_Fig. 2). Although sequence changes necessarily accompany these structural perturbations and may also affect RNA-binding motifs, recovery of repression without restoring the original G-tract supports a structure-dependent component of the genetic variant effect.

The predicted *MAX* structure is also positionally constrained and evolutionarily conserved. Altering the spacing between the G-tract and complementary C-tract changed predicted ΔG (S_Fig. 3). The native 5-nt spacing is conserved in 40 of 41 mammalian species in which both tracts are fully conserved (S_Table 1), and these sequences have predicted ΔG ≤ - 6.3 kcal/mol. The region also shows strong constraint across 470 mammals (phyloP: G-tract, 2.53; C-tract, 4.39; G-tract-AG, 2.54) (6,12). Together, these observations are consistent with evolutionary constraint on sequence features that support splicing repression and predicted local structural stability, although conservation alone cannot distinguish selection on RNA structure from that on overlapping sequence-dependent functions.

About 6 million G-tract-AG signatures occur in the human genome (6), most of which are not detectably used as annotated splice sites. Of the signature sites in human genes, 27% have predicted ΔG below - 4 kcal/mol (Fig. 1C), corresponding to an estimated 1.6 million G-tract-AG signatures genome-wide that fall within the structurally stable range associated with repression of cryptic splicing. The relevance of this mechanism to genetic-variant interpretation is further suggested by thousands of variants in GWAS, ClinVar, and HGMD that disrupt G-tracts within these signatures. Across these datasets, 39–50% of affected 3′SSLs showed variant-associated increases in predicted ΔG (Fig. 2D). One example is *G6PD* c.392G>T (rs137852341), a coding variant associated with G6PD deficiency (13). The T variant disrupts the G-tract and increases predicted ΔG (S_Fig. 4), raising the testable possibility that altered cryptic splicing contributes to its functional consequences in addition to the G131V amino acid substitution (13). More broadly, these observations nominate disruption of local RNA structural repression as a candidate mechanism for functional effects of a subset of trait- or disease-associated variants, including variants whose consequences may not be fully captured by protein-coding annotation or conventional splice-site prediction.

Together with our previous evidence that G-tracts predominantly inhibit splicing after the first catalytic step (6), and structural studies showing that 3′ splice-site positioning and conformational remodeling are required for exon ligation (14–16), these findings suggest a model in which excessive local RNA stability may impede productive second-step progression. Such structural repression could complement earlier regulation by G-tract-binding factors such as hnRNP H1/F (8,17). We propose that G-tract-disrupting SNVs can weaken this barrier and thereby expose cryptic 3′ splice sites to productive splicing. Direct structural and spliceosomal measurements will be required to test this model and determine whether analogous variant-sensitive structural mechanisms operate at non-G-tract cryptic sites.

Our results identify disruption of predicted local RNA structural stability as a candidate mechanism linking genetic variation to cryptic splicing at a widespread class of G-tract-associated 3′ splice sites (Fig. 2E). This mechanism is distinct from variant effects that act primarily by strengthening canonical splice-site sequence motifs and provides a potential explanation for genetic effects that are poorly captured by conventional splice-site scores. Integrating primary sequence, local RNA structural potential, and RNA-binding-protein context may therefore improve mechanistic interpretation and prediction of variant-induced cryptic splicing, including disease-associated variants whose functional consequences remain unresolved.

## MATERIALS AND METHODS

### MaxEnt splice site scoring and minimum free energy (ΔG) prediction:

MaxEnt scores for all 3′SS or 3′SS-like sequences (23 nucleotides: 20 nt intronic plus 3 nt exonic) were calculated using the open-source MaxEntScan algorithm (11). Unless otherwise stated, minimum free energy (ΔG) values for the same 23-nt RNA sequences were predicted using RNAfold from the ViennaRNA package (18,19). These ΔG values therefore represent computational predictions of local thermodynamic stability rather than direct measurements of RNA structure.

### Index of 3′ splice site usage:

The index of 3′SS usage was calculated as described previously (6,20): 1 minus the ratio of intronic to exonic RNA-seq reads within 25-nt windows flanking the intron-exon junction, with values approaching 0 indicating little detectable splicing and values approaching 1 indicating high splice-site usage. Human total-blood RNA-seq data were obtained from the Framingham Heart Study, and G-tract-AG 3′SSs were identified as described previously (6). For the initial correlation analyses, 85 G-tract-AG sites containing three consecutive guanosines [(G)_3_] were selected from our previous analysis of 100 participants (6); only sites at which the G-tract was associated with reduced splice-site usage were included. H-variant effect sizes were subsequently analysed in 3,424 FHS participants using an additive model. Participants were grouped by genotype at SNVs directly disrupting the G-tract: G/G (alternative-allele dosage = 0), G/H (dosage = 1), and H/H (dosage = 2), where H denotes A, C, or T. For annotated rat 3′SSs in S_Fig. 1B, the usage index was calculated from previously reported nuclear RNA-seq data (21). Relationships between mean 3′SS usage and MaxEnt score or ΔG were assessed by linear regression.

### Plasmid construction, cell culture and transfection:

The MAX wildtype splicing reporter and its mutants were constructed using the Gibson Assembly Master Mix (New England Biolabs) as described (6). All plasmid constructs were verified by DNA sequencing.

Human embryonic kidney HEK293T cells, previously verified to be free of mycoplasma contamination (6), were maintained in Dulbecco's modified Eagle medium (Thermo Fisher Scientific) supplemented with 10% fetal bovine serum and 1% penicillin-streptomycin-glutamine at 37 °C in a humidified incubator with 5% CO_2_. Cells were seeded in 6-well plates and transfected with 1 μg minigene plasmid DNA using Lipofectamine 3000 (Invitrogen) according to the manufacturer's protocol. Cells were harvested 24–36 h after transfection for RNA extraction.

### RNA extraction, cDNA synthesis and RT-PCR:

Total RNA was extracted using TRIzol reagent (Invitrogen) following the manufacturer's instructions. RNA quality and concentration were assessed using a NanoDrop spectrophotometer. Reverse transcription was performed with 1 μg of RNA using SuperScript III reverse transcriptase (Invitrogen) and T7Dup10 as primer in a 10 μl reaction (primer sequence: 5′-TAATACGACTCACTATAGGGCAAAGGACTCAAAGAACCTCTG-3′). Semi-quantitative RT-PCR was performed using 1 μl of cDNA in a 24 μl PCR reaction with the following primers, DUP8a: 5′-GACACCATGCATGGTGCACC-3′ and T7: 5′-TAATACGACTCACTATAGGG-3′. PCR products were resolved on 2% agarose gels stained with ethidium bromide, visualized under UV illumination, and quantified using ImageJ. Exon inclusion level was calculated as the percentage of the band intensity of middle exon-included product over the total intensity of spliced products, normalized by product nucleotide lengths.

### Evolutionary conservation analysis:

Sequence alignments of the 3′SS region of *MAX* exon 4 across species were obtained from the UCSC Genome Browser MultiZ alignment of 100 vertebrates (12). PhyloP conservation scores for SNVs and sequence motifs were obtained from the UCSC 470-mammal conservation track as described previously (6,12).

### Statistical analysis:

Linear regression was used to assess quantitative relationships between 3′ splice-site usage and MaxEnt score or ΔG; R^2^ and *p*-values are reported in the figures, with p < 0.05 considered statistically significant. For FHS analyses, alternative-allele dosage (0, 1, or 2) was modelled as a continuous predictor of 3′AG usage. Covariates included sex, blood cell counts, batch, the top five genotype-based principal components (PCs) to account for population structure, and the top 15 transcriptome-based PCs to adjust for additional sources of expression variation. Statistical significance for SNV associations was assessed using Bonferroni correction for multiple SNVs mapping to the same 3′AG site. Enrichment of negative MaxEnt scores or ΔG < −4 kcal/mol between G-tract-AG sites and annotated splice sites, and enrichment of SNV-associated ΔG increases relative to control sequence sets, were assessed by hypergeometric tests. Net ΔG changes produced by G-tract substitutions were tested by one-sample t-tests against a null mean change of 0.

## Supplementary Material

1 **1. Supplementary Table 1.** Evolutionary conservation of the MAX 3′ splice-site RNA structure across mammalian species.
**Figure S1**. Relationship of MaxEnt score and predicted ΔG to 3′AG usage at G-tract-AG 3′ splice sites across genotypes of 100 FHS participants.
**Figure S2**. Additional G-tract-AG and mutant splicing reporters showing structure-associated changes in 3′ splice-site usage.
**Figure S3**. Effects of spacing between stem-forming motifs on predicted RNA structural stability.
**Figure S4**. Disease-associated *G6PD* c.392G>T variant and predicted local 3′ splice-site structural change.

## Figures and Tables

**Figure 1.**
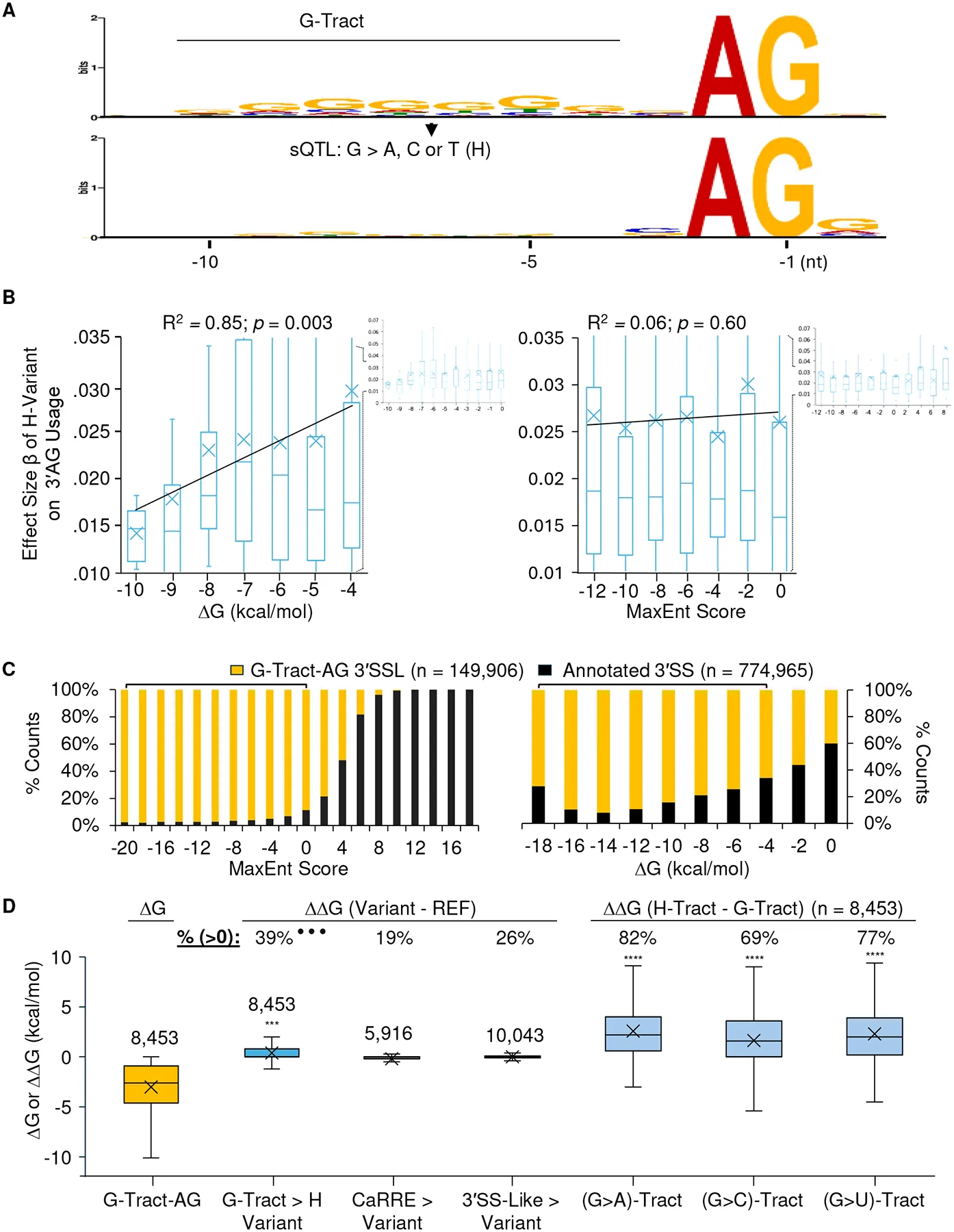
Relationships among local RNA structural stability, G-tract-disrupting variants, and 3′ splice-site activity. (**A**) Enriched sequence motifs within G-tract-AG signatures in non-coding regions identified by MEME analysis of 5,529 signature loci harbouring sQTLs within G-tracts positioned −10 to −3 nt upstream of the 3′AG. Motif E-values were 1.7 × 10^−700^ (n = 5,516) and 8.0 × 10^−227^ (n = 5,527). (**B**) Mean effect size (β) of H-variant-associated enhancement of 3′AG usage plotted against predicted ΔG (left) or MaxEnt score (right). H denotes a G-tract-disrupting A, C, or T allele. Effect sizes represent the additive contribution per H allele relative to the G allele across G/G (dosage 0), G/H (1), and H/H (2) genotypes. n = 1,018 SNV-AG pairs in total analysed in 3,424 FHS participants. The model was adjusted for sex, blood cell counts, batch, the top five genotype-based principal components, and the top 15 transcriptome-based principal components. Data are mean ± SEM for sites with H-variant ΔG < −4 kcal/mol. The 1,018 SNV-AG pairs were grouped by ΔG or MaxEnt score as indicated; points/boxes show the distribution within each bin. Regression was performed across the bin means. The correlation remains with different bin sizes. (**C**) Relative distributions of MaxEnt scores (left) and predicted ΔG values (right) for human G-tract-AG 3′ splice-site-like loci (3′SSLs; n = 149,906) in human genes and annotated human 3′SSs (n = 774,965; Ensembl BioMart v16). (**D**) Net ΔG changes at G-tract-AG sQTL sites (n = 8,453) produced by observed G-tract-disrupting SNVs or by in silico substitution of G-tracts with A-, C-, or U-tracts. CA-rich CaRRE sites (n = 5,916) and random 3′SS-like sites lacking G-tracts (n = 10,043) are shown as controls. ***p = 1.10 × 10^−136^ and ****p < 1.16 × 10^−458^, one-sample t-test against a null mean ΔG change of 0; ^●●●^*p* < 2.94 × 10^−69^, hypergeometric test. REF denotes the reference nucleotide(s). In box plots, the horizontal line indicates the median, × indicates the mean, box boundaries indicate the 25th and 75th percentiles, and whiskers indicate the plotted lower and upper limits.

**Figure 2.**
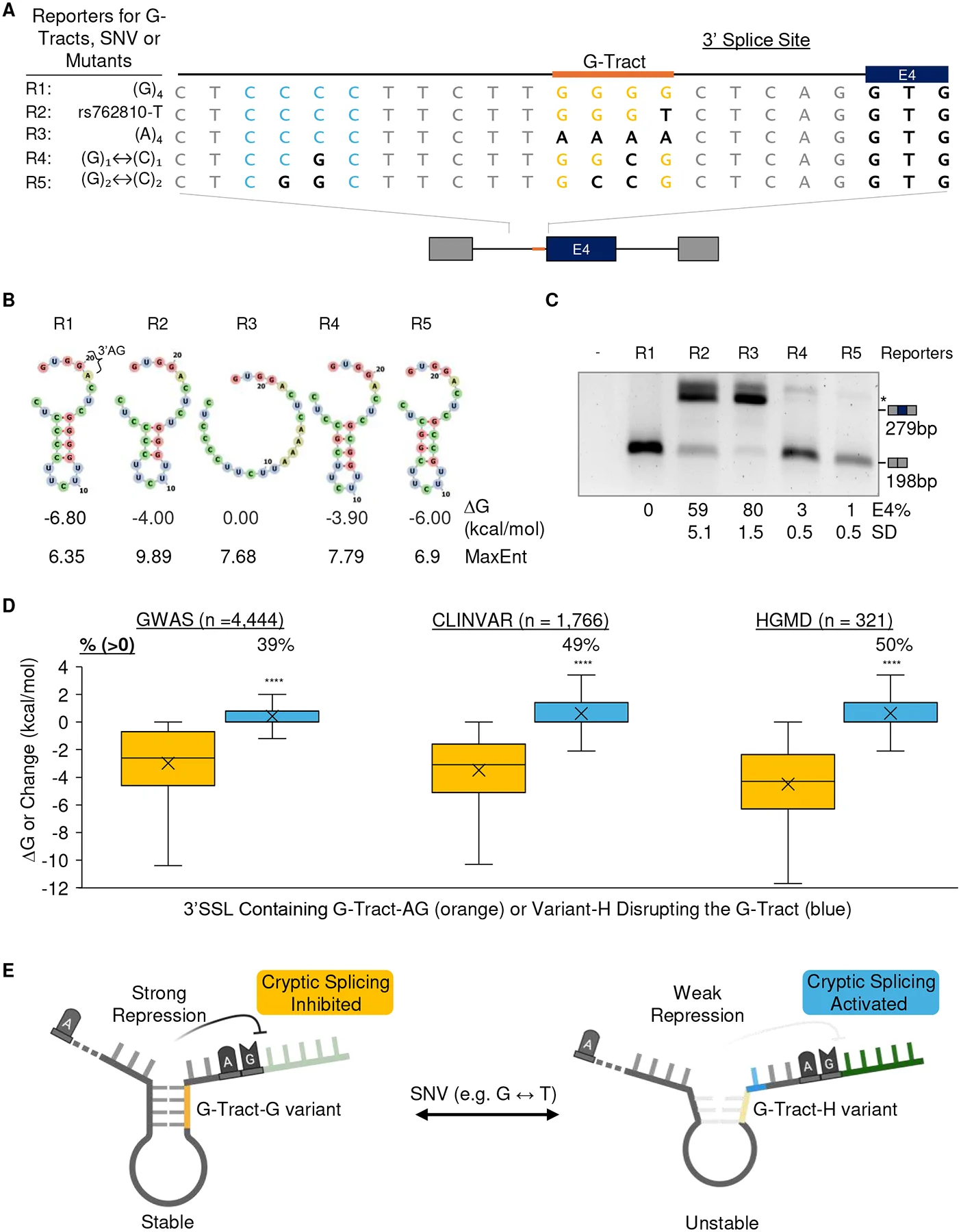
Local RNA structural stability contributes to repression of G-tract-AG cryptic 3′ splice sites and is disrupted by genetic variants. (**A**) Sequence alignment of the 3′ splice-site regions of five *MAX* exon 4 minigene reporters (R1-R5), highlighting the G-tract, complementary C-tract, 3′AG, and engineered substitutions. (**B**) RNAfold-predicted secondary structures, corresponding ΔG values, and 3′SS MaxEnt scores for the five reporters. (**C**) Agarose-gel analysis of RT-PCR products from reporters expressed in HEK293T cells. The asterisk denotes a heteroduplex of splice products verified by DNA sequencing. (**D**) ΔG changes produced by G-tract-disrupting variants at G-tract-AG sites represented in GWAS, ClinVar, and HGMD datasets. ****p < 2.92 × 10^−85^, one-sample t-test against a null mean change of 0. Box plots are defined as in Fig. 1D. (**E**) Model for the RNA structure-dependent repression of cryptic 3′ splice sites. A stable local stem-loop is associated with repression of cryptic 3′SS usage, whereas a G-tract-disrupting SNV can destabilize the structure and promote cryptic splicing. Compensatory restoration of predicted structural stability re-establishes repression, supporting a causal contribution of local RNA structural stability in the reporter system.

## Data Availability

The whole genome sequencing (WGS) and RNA sequencing (RNA-Seq) data from the Framingham Heart Study are available at the dbGaP database under accession code phs000007.v34.p15 [https://www.ncbi.nlm.nih.gov/projects/gap/cgi-bin/study.cgi?study_id=phs000007.v34.p15] (22). These data are available under restricted access to ensure confidentiality and protect participant privacy. Access can be obtained by submitting an ancillary study proposal and obtaining IRB approval. Timelines for the approval process range from 3–6 weeks. Researchers must first obtain an eRA Commons account, which is typically set up within 3–7 business days if the applicant’s institution is already registered with NIH (institutional registration may add an additional 2–4 weeks). Our published raw RNA-Seq reads for the verification of HEK293T identity are freely available from the NCBI Sequence Read Archive with accession numbers SRX32695245, SRX32695246 and SRX32695247 (8). Raw RNA-Seq reads for the rat GH_3_ cell samples are available under accession numbers SRX33583299, SRX33583300 and SRX33583303 (21,23). The source code for splice site usage index is released under an open source license compliant with OSI (http://opensource.org/licenses) with accession #31820986, at https://doi.org/10.6084/m9.figshare.31820986 (24). The gel images and supplementary files have been deposited in figshare at https://doi.org/10.6084/m9.figshare.32383353 with accession number 32383353.
