# Supplementary material for: Genetic Variation Disrupts RNA Structural Repression of Cryptic Splice Sites
Source: Res Sq. 2026 Oct 7:rs.3.rs-11246309. Preprint. [Version 1] doi: 10.21203/rs.3.rs-11246309/v1 (PMC13692680; doi:10.21203/rs.3.rs-11246309/v1)
Supplement: Supplementary file 1 [file NIHPPRS11246309V1-supplement-1.pdf]

Supplementary Table/Figures

S_Table 1. Evolutionary conservation of the stem-loop structure at the upstream 3′ splice site of *MAX* exon 4. Shown is the multiZ sequence alignment from the UCSC Genome Browser, with RNAfold-predicted ΔG values ≤ -6.3 kcal/mol shaded in brown (input sequence from C- to G-tract). Note: the CCCC and GGGG motifs, their gap size and overall structural stability are conserved across most mammalian species. Motif- or gap size-disrupting nucleotides are in light blue.

Organism	3'SS Sequence	ΔG
Human	---------acc--tct cccc -----t--------tc-tt gggg ctcaggtgc--	-6.3
Human_SNV-T	---------acc--tct cccc -----t--------tc-tt gggt ctcaggtgc--	-4.0
Brush-tailed rat	---------ctc--gct ccca agaagg--------cc-tt gggg ctcaggtgc--	-8.8
Bushbaby	---------atc--tct cccc -----t--------tt-tg gggg cgtaggtgc--	-7.7
Cat	---------atc--tct cccc -----t--------tc-tg gggg ctcaggtgc--	-7.7
Black flying-fox	---------att--tct cccc -----t--------tt-tg gggg ctcaggtgt--	-7.7
Megabat	---------att--tct cccc -----t--------tt-tg gggg ctcaggtgt--	-7.7
Tenrec	---------att--tct cccc -----g--------ctgtc gggg tgcaggtgc--	-7.2
Pika	----------------t cccc -----g--------cc-tc gggg ctcaggtgt--	-6.9
Little brown bat	---------atc--tct cccc -----c--------tc-tt gggg cttaggtgc--	-6.7
Chimp	---------acc--tct cccc -----t--------tc-tt gggg ctcaggtgc--	-6.3
Gorilla	---------acc--tct cccc -----t--------tc-tt gggg ctcaggtgc--	-6.3
Orangutan	---------atc--tct cccc -----t--------tc-tt gggg ctcagatgc--	-6.3
Gibbon	---------acc--tct cccc -----t--------tc-tt gggg ctcaggtgc--	-6.3
Rhesus	---------acc--tct cccc -----t--------tc-tt gggg ctcaggtgc--	-6.3
Crab-eating macaque	---------acc--tct cccc -----t--------tc-tt gggg ctcaggtgc--	-6.3
Baboon	---------acc--tct cccc -----t--------tc-tt gggg ctcaggtgc--	-6.3
Green monkey	---------acc--tct cccc -----t--------tc-tt gggg ctcaggtgc--	-6.3
Marmoset	---------atc--tgt cccc -----t--------tc-tt gggg ctcaggtgc--	-6.3
Squirrel monkey	---------atc--tgt cccc -----t--------tc-tt gggg ctcaggtgc--	-6.3
Chinchilla	---------ctc--tct cccc -----t--------tc-ct gggg ctcgggtgc--	-6.3
Pig	---------ctctctct cccc -----t--------tc-tt gggg ctcaggcgc--	-6.3
Alpaca	---------ctc--tct cccc -----t--------tc-tt gggg ctca-gtgc--	-6.3
Bactrian camel	---------ctc--tct cccc -----t--------tc-tt gggg ctca-gtgc--	-6.3
Dolphin	---------ctctctct cccc -----t--------tc-tt gggg ctcaggtgc--	-6.3
Killer whale	---------ctctctct cccc -----t--------tc-tt gggg ctcaggtgc--	-6.3
Tibetan antelope	---------ctcgctct cccc -----t--------tc-tt gggg ctcaagtgt--	-6.3
Cow	---------ctcgctct cccc -----t--------tc-tt gggg ctcaggtgt--	-6.3
Sheep	---------ctcgctct cccc -----t--------tc-tt gggg cccaagtgt--	-6.3
Domestic goat	---------ctcgctct cccc -----t--------tc-tt gggg ctcaagtgt--	-6.3
White rhinoceros	---------atc--tct cccc -----t--------tc-tt gggg ctcaggtgc--	-6.3
Dog	---------atc--tct cccc -----t--------tc-tt gggg ctcaggtgg--	-6.3
Ferret	---------atc--tct cccc -----t--------tc-ct gggg ctcaggtgc--	-6.3
Panda	---------atc--tct cccc -----t--------tc-tt gggg ctcaggtgc--	-6.3
Pacific walrus	---------atc--tct cccc -----t--------tc-tt gggg ctcaggtgc--	-6.3
Weddell seal	---------atc--tct cccc -----t--------tc-tt gggg ctcaggtgc--	-6.3
Big brown bat	---------atc--tct cccc -----t--------tc-tt gggg cttgggtgc--	-6.3
David's myotis (bat)	---------atc--tct cccc -----t--------tc-tt gggg cttaggtgc--	-6.3
Hedgehog	---------att--tct cccc -----t--------tc-tt gggg cttagatgc--	-6.3
Shrew	---------atc--tct cccc -----t--------tt-tt gggg ctcaggtgc--	-6.3
Manatee	---------atc--tct cccc -----t--------tc-tt gggg ctcaggtgc--	-6.3
Cape golden mole	---------att--tct cccc -----t--------ct-tt gggg cccaggtgc--	-6.3
Armadillo	---------atc--tct cccc -----t--------tc-tt gggg ctcaggtgc--	-6.3

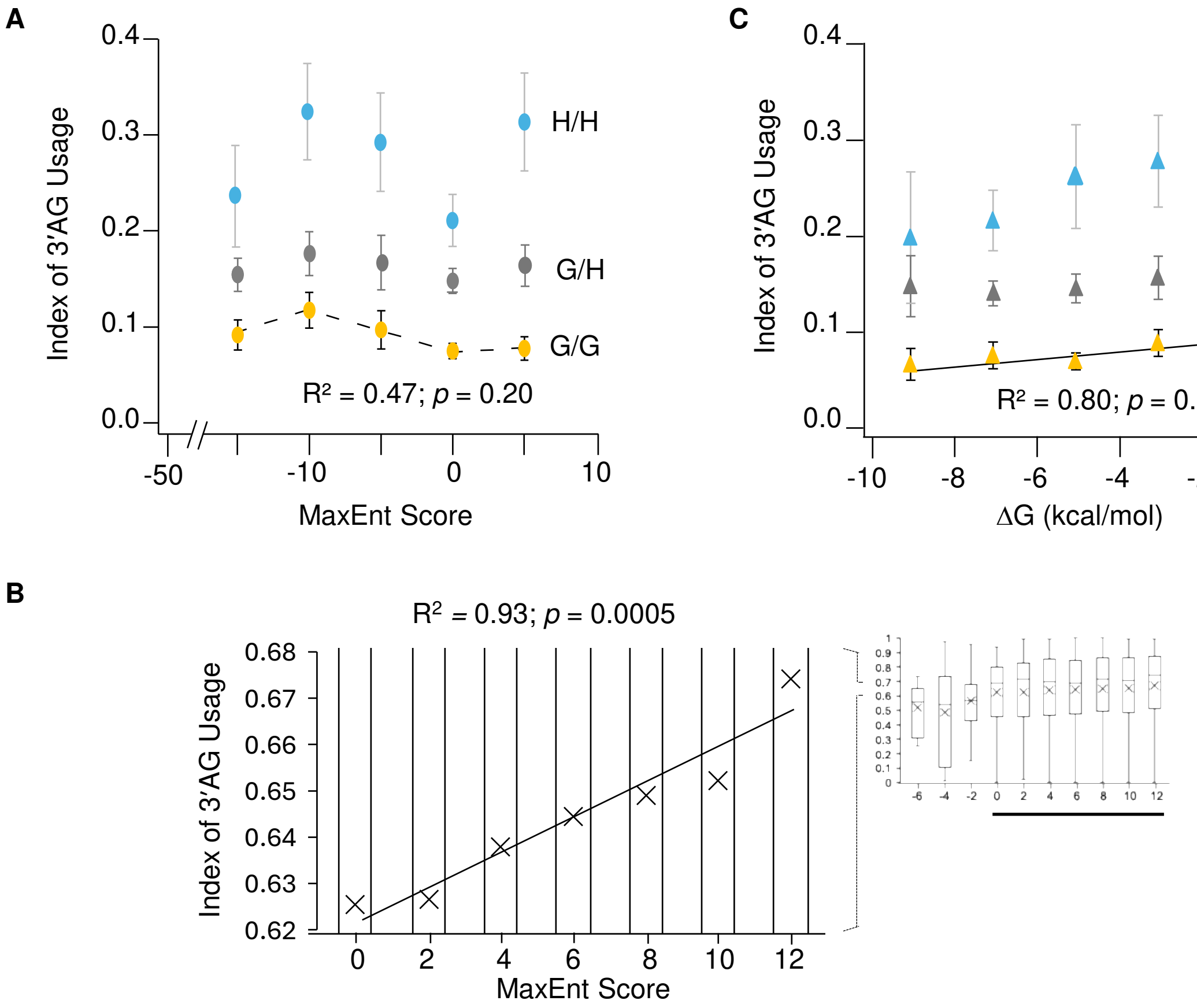

S_Figure 1. Relationship of MaxEnt score and ΔG to 3′ AG usage at G-tract-AG 3′ splice sites across genotypes. (**A**) Plot of the relationship between mean MaxEnt score (± SEM, n = 85 3′SSs) and the index of 3′ AG usage for G-tract-AG 3′ splice sites containing $(G)_3$ motifs, identified from the Framingham Heart Study RNA-seq data. MaxEnt scores were grouped into 5-unit bins inclusive of the upper bound, with the leftmost bin defined as $-50 < X \leq -15$. In addition to the G/G genotype, the corresponding G/H (heterozygous disruption; H = G-tract-disrupting alleles A, C, or T) and H/H (homozygous disruption) genotypes at SNV loci are shown to illustrate variant effects on splice-site usage. (**B**) Relationship between the index of 3′ splice-site (3′SS) usage and MaxEnt score for annotated 3′SSs in rat GH_3 pituitary cells (n = 4,680), grouped into 2-unit bins inclusive of the upper bound. (**C**) Plot of the relationship between mean ΔG (± SEM) and the index of 3′AG usage for the same set of G-tract-AG sites analyzed in panel A. ΔG values were calculated from 23nt 3′SS sequences and grouped into 2-unit bins inclusive of the upper bound, with the leftmost bin defined as $-12 < X \leq -8$ kcal/mol. The corresponding genotypes of other SNV loci (alleles with non-G variants, A, T or C, called H-variant collectively) are also shown to illustrate the effects of G-tract disruption on splicing activity. The correlation remains with different bin sizes.

GRK5 *PRMT5* *PAX8*

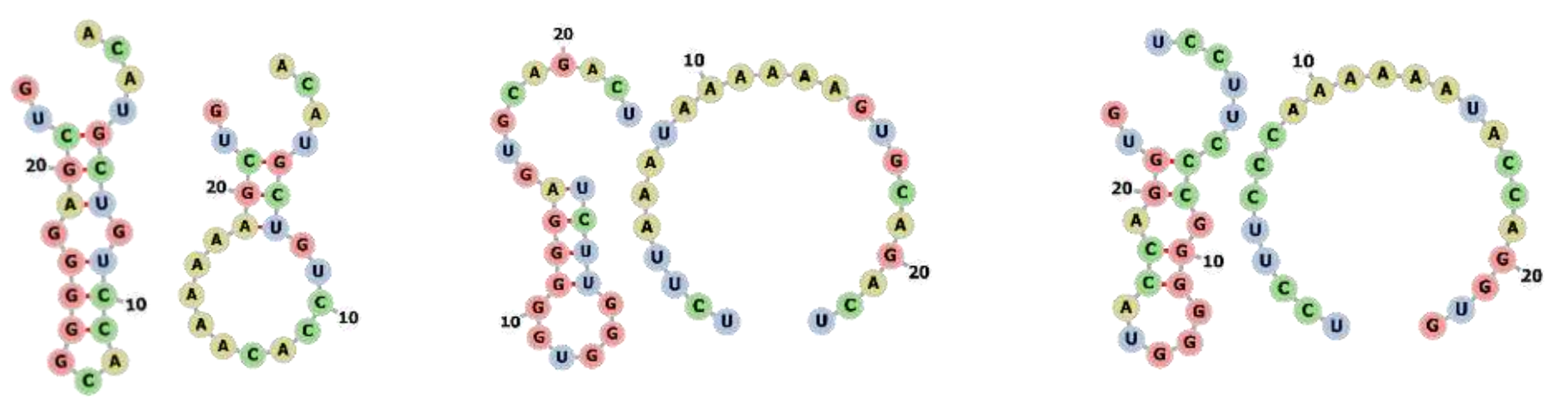

	GRK5		*PRMT5*		*PAX8*	
ΔG (kcal/mol)	-6.80	-1.60	-1.00	0.00	-3.90	0.00
MaxEnt Scores	-6.04	-0.28	-6.01	-2.39	2.95	4.50
% Exon Inclusion	0.90	11.90	1.40	11.3	3.50	96.80

S_Figure 2. Additional examples of 3′SS RNA secondary structures with corresponding splicing outcomes of G-tract-AG reporters from the *GRK5*, *PRMT5*, and *PAX8* genes. For each gene, predicted structures are shown for the wild-type G-tract sequence (left) and corresponding A-tract substitution mutant (right). ΔG values (kcal/mol), MaxEnt scores, and exon inclusion percentages are indicated below each structure pair. A-tract substitution consistently increased ΔG values (*GRK5*: -6.80 to -1.60 kcal/mol; *PRMT5*: -1.00 to 0.00 kcal/mol; *PAX8*: -3.90 to 0.00 kcal/mol), indicating destabilization of the 3′SS RNA structure, and was accompanied by corresponding increases in exon inclusion (*GRK5*: 0.90% to 11.90%; *PRMT5*: 1.40% to 11.30%; *PAX8*: 3.50% to 96.80%), consistent with an inverse relationship between predicted local structural stability and 3′ splice-site usage. In contrast, MaxEnt scores did not display a consistent directional trend among genes. Together, these reporter analyses support an important contribution of predicted local RNA structural stability to repression at G-tract-AG sites. Exon inclusion data are from Sohail et al. *BMC Genomics* 2014 study.

Gap (nt)	Gap	ΔG (kcal/MOL)
8	ctccccttcttctcggggagGTG	-11.6
7	ctccccttcttctggggcagGTG	-8.00
6	ctccccttcttcggggtcagGTG	-6.60
5	**ctccccttcttggggctcagGTG**	-6.80
4	ctccccttctggggtctcagGTG	-7.10
3	ctccccttcggggttctcagGTG	-5.2

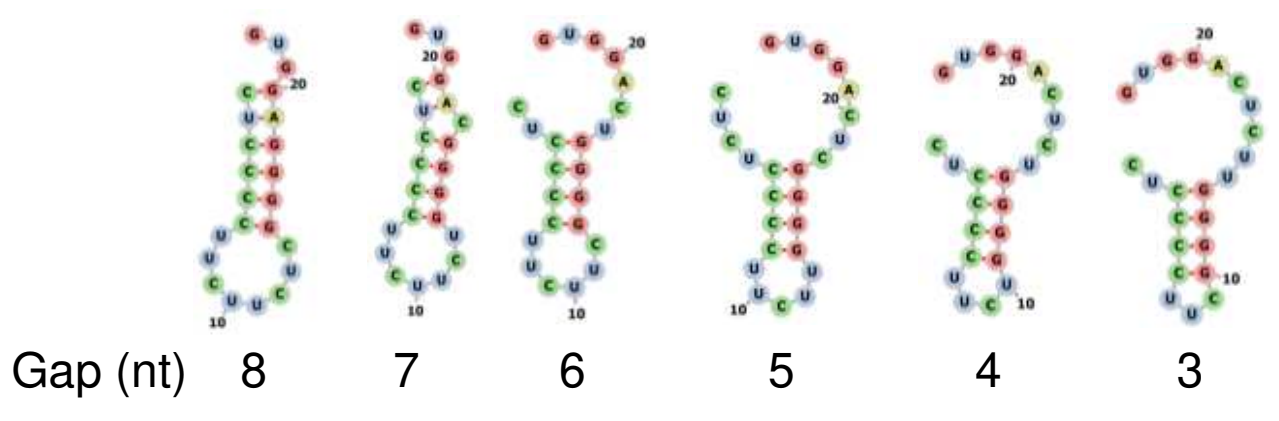

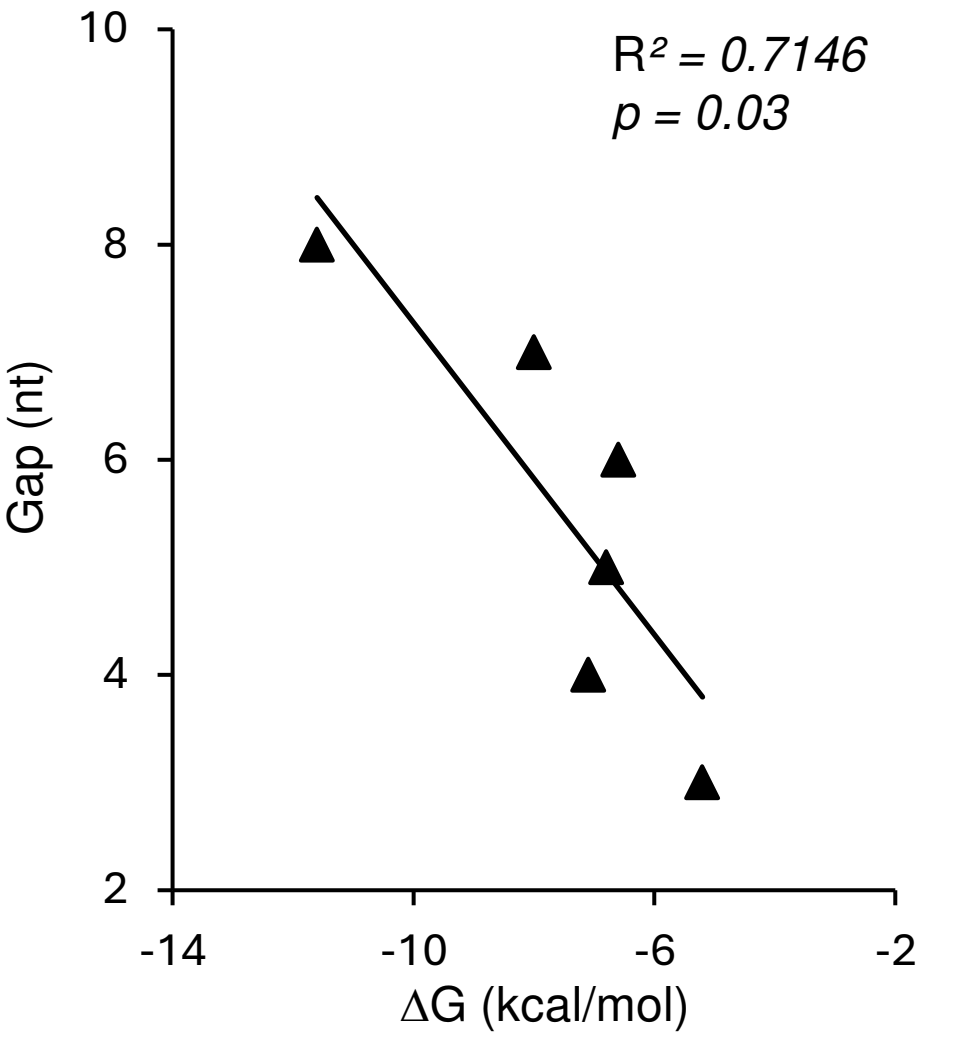

S_Figure 3. Spatial gap between the G-tract and its complementary C-tract influences stem-loop stability. (Left) Six *MAX* exon 4 3′SS sequence variants with progressively increasing spatial gaps between the G-tract (orange) and complementary C-tract (blue), together with corresponding RNAfold-predicted ΔG values (kcal/mol) of the 23nt 3′SS. Variant sequences are computationally generated with decreasing gap sizes, with the native *MAX* exon 4 3′SS sequence in bold. Predicted RNA secondary structures for each variant are shown below the sequences. (Right) Scatter plot of gap size (nt) versus RNAfold-predicted ΔG (kcal/mol) across all six variants, demonstrating a significant correlation. Larger spatial gaps between the G-tract and complementary C-tract are associated with more negative ΔG values, indicating increased stem-loop stability. R^2 and p-values are from linear regression analysis.

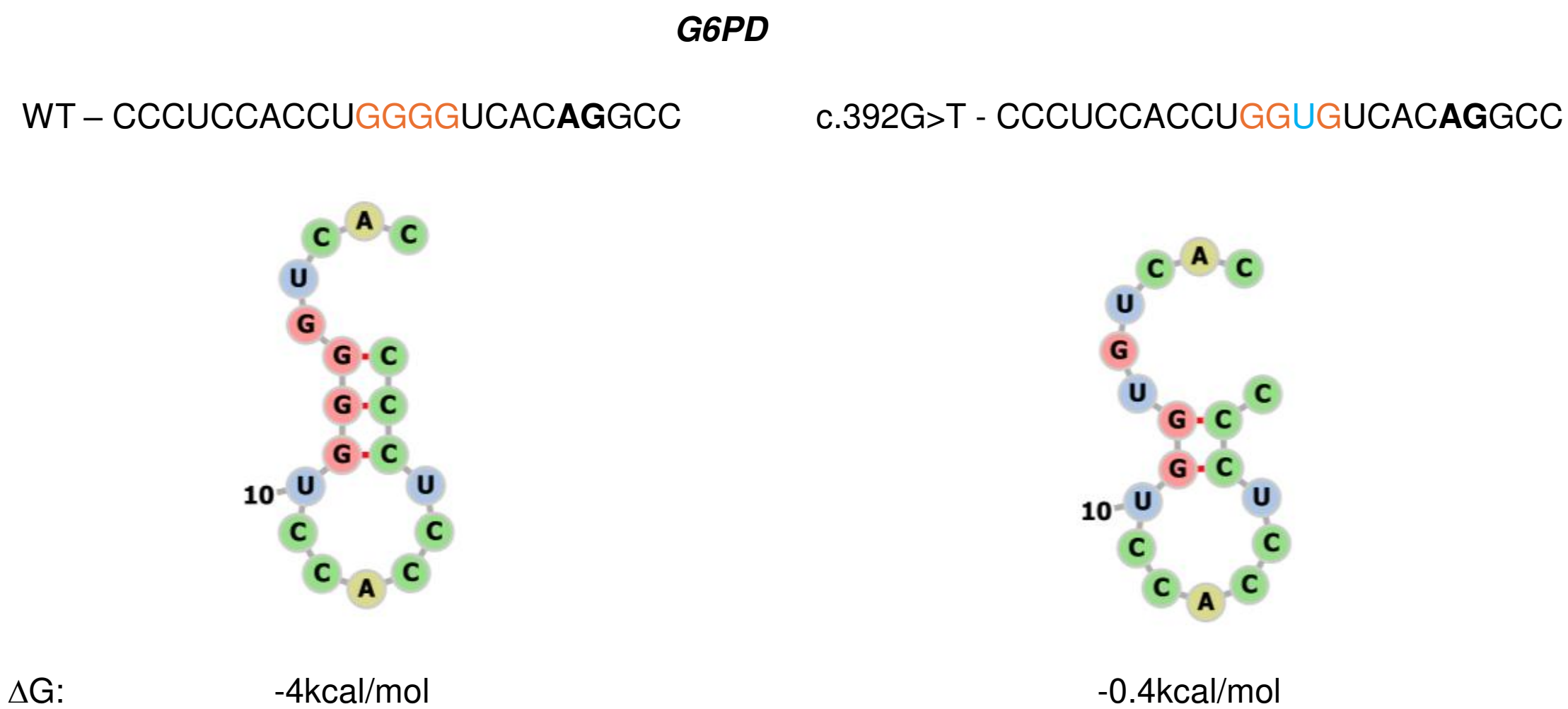

S_Figure 4. Predicted destabilization of local 3′ splice-site RNA structure by the disease-associated G6PD c.392G>T (rs137852341) variant. The variant occurs within the coding region of *G6PD* and results in the G131V amino acid substitution associated with G6PD deficiency (Li et al., *Human Genomics*, 2023). RNAfold predicts that the variant increases local ΔG from −4.0 to −0.4 kcal/mol, consistent with destabilization of the predicted RNA structure upstream of the 3′AG.
